# Quality of life and health status of Indonesian women with breast cancer symptoms before the definitive diagnosis: A comparison with Indonesian women in general

**DOI:** 10.1371/journal.pone.0200966

**Published:** 2018-07-19

**Authors:** Hari Setyowibowo, Fredrick Dermawan Purba, Joke A. M. Hunfeld, Aulia Iskandarsyah, Sawitri S. Sadarjoen, Jan Passchier, Marit Sijbrandij

**Affiliations:** 1 Department of Clinical, Neuro and Developmental Psychology, VU University, Amsterdam, The Netherlands; 2 Department of Educational Psychology, Faculty of Psychology, Universitas Padjadjaran, Jatinangor, Indonesia; 3 Department of Psychiatry, Section Medical Psychology and Psychotherapy, Erasmus MC University Medical Center, Rotterdam, The Netherlands; 4 Department of Developmental Psychology, Faculty of Psychology, Universitas Padjadjaran, Jatinangor, Indonesia; 5 Department of Clinical Psychology, Faculty of Psychology, Universitas Padjadjaran, Jatinangor, Indonesia; Hong Kong Polytechnic University, HONG KONG

## Abstract

**Objectives:**

Breast cancer (BC) is prevalent in low and middle-income countries (LMICs) where the majority of cases are diagnosed in late stages. The aims of this study were: (1) to assess quality of life (QOL) and health status of Indonesian women with BC symptoms before definitive diagnosis; (2) to compare QOL and health status between women with BC symptoms before definitive diagnosis and Indonesian women in general; (3) to evaluate the association between demographic variables (age, residence, social economic status and education level) and QOL within the Indonesian women with BC symptoms before definitive diagnosis.

**Methods:**

We used WHOQOL-BREF to measure QOL and EQ-5D-5L for health status. Multivariate analysis of covariance (MANCOVA) was used to compare QOL and health status between women with BC symptoms and women from the general Indonesian population in order to control for confounders. Regression analyses were used for testing the association between the demographic variables, QOL, and health status.

**Results:**

In comparison with the data from the women from the general population (n = 471), the women with BC symptoms (n = 132) reported lower QOL, especially in physical and psychological domains. They also reported more problems in all dimensions of health status. Higher education and monthly income were positively associated with QOL and health status among the women with BC symptoms.

**Conclusion:**

Before receiving a definitive diagnosis, women who visit hospitals with symptoms of BC, report a lower QOL and health status than women in general. Our results suggest that healthcare providers should provide targeted strategies for women with BC symptoms to improve their QOL.

## Introduction

Breast cancer (BC) is the most frequently diagnosed malignant tumor among women in both high-income countries (HICs) and low and middle-income countries (LMICs) [1]. The incidence of BC in LMICs is lower than for HICs, but mortality rates in LMICs are higher than in HICs because of advanced-staged diagnosis and inadequate access to care [2]. The mortality rates have been decreasing in many HICs since around 1990 due to early detection and improved treatment [2]. In Indonesia, BC continues to be the most common malignancy in women with an incidence rate of 40.3 percent and a mortality rate of 16.6 percent per 100,000 people [3].

The diagnostic process of BC and its treatments are often associated with negative effects that can lead to lower quality of life (QOL) [4, 5]. Consequently, the current intervention of BC should not only focus on illness control but also to maintain and improve QOL of women with BC. Throughout the process of hospital care, from diagnosis to treatment, the BC examinations and treatments affect the physical, psychological and social aspects of the life of a woman, which can significantly reduce her QOL, increase psychological distress [6], and uncertainty [7], negatively affect her body image and sexuality [8], illness perception [9], and increase unmet health needs [10]. Therefore, the information about QOL is crucial at every stage of the BC trajectory. However, most investigations explore various issues after definitive diagnosis, e.g., QOL in women with BC during treatment [9, 11, 12] and QOL in BC survivors [13–15].

To our knowledge, no previous investigation was conducted among the women before the definitive diagnosis in Indonesian women. This stage is essential because some psychosocial problems might already occur when women find abnormalities in their breasts. One of the issues may be the uncertainty about having or not having the disease and the future treatment process. The diagnosis of BC may have many consequences, not only related to life expectancy but also to QOL. Some women with BC in Indonesia have a belief that BC is an incurable and deadly disease [16]. On the other hand, despite having discovered the symptoms of BC, some women may assume that they have no severe health problems. Given the interrelation between demographic characteristics, QOL and health status in general [17–20], we also evaluated which demographic attributes might contribute to a lower QOL and health status in the women with BC symptoms.

No studies have yet been published that compared QOL and health status between undiagnosed women with BC symptoms and women in the general population in Indonesia. Therefore, the aims of this study were: (1) to assess QOL and health status of Indonesian women with BC symptoms before receiving a definitive diagnosis; (2) to compare QOL and health status between women with BC symptoms and Indonesian women in general; (3) to evaluate the association of socio-demographic factors with QOL and health status of Indonesian women with BC symptoms before the definitive diagnosis. The current study that investigated QoL and health status for women with BC symptoms before definite diagnosis may serve as a bridge for future studies to explore whether psychosocial concerns such as impaired body image and unmet needs should be taken into account for women with BC symptoms before definitive diagnosis.

## Methods

### Participants

This study consisted of two groups of participants: 1) Indonesian women with BC symptoms who already consulted the hospital but yet without a definitive diagnosis (BC Symptoms Group) and (2) Indonesian women from the general population (General Population Group).

#### BC symptoms group

Participants were recruited from four district hospitals in West Java, Indonesia. They were new patients (outpatients) who visit the hospital with breast symptoms, which make them suspects of having BC, but yet without a definitive diagnosis. The following inclusion criteria were used: women with age 18 years and above, an adequate command of the Indonesian language (Bahasa Indonesia) and no major psychiatric disorder. The last criterion was determined by checking the medical record on a consultation history/record with the Psychiatric Department. Patients who have been seen by a psychiatrist were excluded from the study.

#### General population group

The data of the comparison group (which will be referred to as "general population" in this manuscript) were women selected from a larger study focused upon the Indonesian general population, in which several questionnaires were tested in a face-to-face setting at the home/office of the interviewer or at the homes of the subjects [21].This study implemented a multi-stage stratified quota sampling method to ensure representativeness with the Indonesian general population, which resulted in 1054 participants being interviewed. Only the female participants with the age of 18 years and above from the aforementioned study were included in the analysis of the present study.

### Instruments

A standard socio-demographic questionnaire was used to collect participants' background data on residence, age, education level and income level.

QOL was measured using the Indonesian version of the WHOQOL-BREF, with a four weeks-time retrospection. WHOQOL-BREF has been utilized in several investigations in the BC populations in Asian countries [22], including Indonesia [9]. This instrument is a self-report questionnaire that consists of 26 items. The internal consistency of the WHOQOL BREF’s domains in the present sample were 0.70, 0.78, 0.57, and 0.75 for physical, psychological, social, and environmental domain respectively. Two items measure QOL and health satisfaction in general. Twenty-four items measure four broad domains: (i) physical health (7 items), e.g., "Do you have enough energy for everyday life?”, (ii) psychological health (6 items), e.g., "How much do you enjoy life?”, (iii) social relationships (3 items), e.g., "How satisfied are you with your personal relationships?” and (iv) environmental (8 items), e.g., "How satisfied are you with the conditions of your living place?” Each item is rated using a 5-point Likert scale with varied wording on each scale depending on the item (for example 1 = very dissatisfied to 5 = very satisfied) [23]. The internal consistency of the WHOQOL BREF domains in the present sample were 0.70, 0.78, 0.57, and 0.75 for physical, psychological, social, and environmental domain respectively.

The health status of the participants was measured by the EQ-5D-5L [24]. This instrument has been used in several BC patient populations around the world [25]. EQ-5D-5L is a generic health-related QOL instrument based on a descriptive system that defines health in terms of five dimensions: mobility (MO), self-care (SC), usual activities (UA), pain/discomfort (PD), and anxiety/depression (AD).

Each dimension has five levels: (1) no problems, (2) slight problems, (3) moderate problems, (4) severe problems, and (5) extreme problems/unable. Therefore, the EQ-5D-5L instrument describes 3125 (5^5^) unique health states. A 1-digit number expresses the level selected for that specific dimension. A specific health state then consisted of a combination of a 5-digit number for the five dimensions. For example, state ‘11111’ indicates ‘no problems on any of the five dimensions’, while state ‘34512’ indicates ‘moderate problems in walking about, severe problems washing or dressing, extreme problems doing usual activities, no pain or discomfort, and slight anxiety or depression’. This descriptive system is followed by a self-rating of overall health status on a visual analogue scale (EQ-VAS) ranging from 0 ("the worst health you can imagine") to 100 ("the best health you can imagine"). EQ-5D-5L has been proven as a valid and reliable questionnaire to be used in Indonesia [26].

### Data collection procedures

#### BC symptoms group

The study was approved by the Health Research Ethics Committee of Dr. Hasan Sadikin General Hospital Bandung. Participants who agreed to participate by means of oral or written consent were asked to complete the following instruments in the hospital: (1) the socio-demographic and medical history form, (2) the WHOQOL-BREF, and (3) the EQ-5D-5L. If they had difficulties in completing the instruments, the interviewers helped them by reading the items out loud and asking the participants to indicate the answers.

#### General population group

The study was approved by the Health Research Ethics Committee, Faculty of Medicine, Universitas Padjadjaran, Indonesia [21]. A representative sample from the Indonesian general population was recruited using multi-stage stratified quota sampling. The interviewers explained the objectives of the study, followed by filling in the informed consent when the participants agreed to participate. Three instruments completed by the participants were: (1) the socio-demographic form: age, sex, income, and education, (2) the WHOQOL-BREF, (3) the EQ-5D-5L. The participants were helped by the interviewers whenever they had problems completing the questionnaires.

### Data analysis

Demographic characteristics were summarized using descriptive statistics, including percentages for categorical data, and means and standard deviations for continuous data. The self-reported health problems obtained from the EQ-5D-5L were presented in percentages of each level of each dimension that was answered positively and then compared between the groups with the Chi-square test. Each participant’s EQ-5D-5L responses then were transformed to a single index score based on the preference of the Indonesian general population, a so-called ‘value set’[21]. For instance, the health state of ‘11111’ corresponds to an EQ-5D-5L index score of 1.00, and ‘22211’, which means ‘slight problems in mobility, self-care and usual activities and no problems in pain/discomfort and anxiety/depression’ leads to a value of 0.69. Mean and standard deviation were calculated for the EQ-VAS, EQ-5D-5L index score and each domain of the WHOQOL-BREF.

For the comparison of the QOL between the two groups, we applied an independent t-test if the data were normally distributed or the Wilcoxon rank-sum test if not normally distributed. Normality was tested using the Shapiro-Wilk test and visual inspection of the histograms. For determining the magnitude of the differences, we calculated the effect size using Cohen's d, and we applied the criteria from Cohen for the interpretation: 0.2–0.5 = small, 0.5–0.8 = medium, >0.8 = large difference [27].

We also applied multivariate analysis of covariance (MANCOVA) with the QOL (WHOQOL-BREF: physical, psychological, social, and environment domain scores) and health status (EQ-5D-5L: EQ-VAS and index score) scores as outcomes and a variable ‘group’ (BC symptoms group vs. general population group) as the predictor in the MANCOVA. Further multiple linear regression analysis was carried out to evaluate whether and if so which socio-demographic variables were significantly affect the QOL and health status scores in the BC symptom group only. Beforehand, a Spearman correlation analysis was done to check whether any significant correlation(s) between each sociodemographic variable with other demographic variables and the outcomes (QOL and health status). We found that ethnicity and religion had no significant correlation to any other variables, therefore they were excluded from the analysis. The following sociodemographic variables: residence (urban/rural), age, level of education (basic: primary school and below/middle: high school/high: all others), and monthly income (below 2 million IDR/2-4 million IDR/above 4 million IDR) were included in the multiple linear regression analysis. Statistical analyses were performed using SPSS-IBM version 21; p-values < .05 were considered statistically significant.

## Results

### Characteristics of participants

The demographic characteristics of women with BC symptoms and the general population are presented in Table 1. The proportion of age and residence subgroups were similar between the two groups. The BC symptom group had a significantly higher level of education and monthly income than the general population group.

**Table 1:** Demographic characteristics of the Breast Cancer (BC) symptoms sample (N = 132) and the general population sample (N = 471).

Characteristic	Level	BC Symptom		General Population^a^		Pearson’s χ^2^ (df)	P-value
		N	(%)	N	(%)		
Age	18–30 years	48	(36.4)	157	(33.3)	0.4263 (2)	0.808
	31–50 years	58	(43.9)	218	(46.3)		
	> 50 years	26	(19.7)	96	(20.4)		
						
Residence	Rural	74	(56.1)	217	(46.1)	4.1196 (1)	0.042
	Urban	58	(43.9)	254	(53.9)		
						
Level of Education^b^	Basic	22	(16.7)	172	(36.5)	18.9003 (2)	<0.001
	Middle	79	(59.8)	222	(47.1)		
	High	31	(23.5)	77	(16.3)		
						
Income/month^c^	Low	60	(45.5)	403	(85.6)	93.2667 (2)	<0.001
	Middle	54	(40.9)	49	(10.4)		
	High	18	(13.6)	19	(4.0)		

^a^based on study Purba, et al., 2017

^b^Basic: primary school and below, Middle: high school, High: all others

^c^Low:2 million IDR, Middle: 2–4 million IDR, High: above 4 million IDR

### QoL and health status in BC symptoms group and general population group

The mean scores of the QOL domains: measured by the WHOQOL-BREF and health status: measured by the EQ-5D-5L are summarized in Table 2. Concerning the QOL, the BC symptoms group showed significantly lower scores (less favorable) on physical and psychological domains than the general population. The effect sizes for these differences were considered as small. For health status, the EQ-VAS and index score of women with BC symptoms were significantly lower than the general population with medium effect sizes for both scores.

**Table 2:** Comparison of means of quality of life (QOL) and health status of the Breast Cancer (BC) symptoms and the general population sample.

		BC symptom		General population		t-statistic^a^	P-value	Effect Size^b^
Aspect	Dimension	Mean	SD	Mean	SD			
Quality of life	Physical health	63.2	13.9	67.9	11.3	4.004	<0.001	0.4
	Psychological health	62.5	15.7	65.2	12.4	2.042	0.042	0.2
	Social relations	62.8	14.4	62.0	13.5	-0.609	0.543	-0.1
	Environment	59.3	12.6	58.4	12.9	-0.729	0.467	-0.1
Health status	EQ-VAS	69.1	20.0	78.6	14.7	6.010	<0.001	0.6
	Index score	0.8	0.2	0.9	0.1	9.066	<0.001	0.9

^a^all degress of freedom (df) = 601

^b^Effect size based on Cohen's d

Table 3 presents the comparison of the EQ-5D-5L self-reported health status of the BC symptom and the general population samples. The proportions of responses for each severity level of problems were significantly different for all dimensions between the two samples. It can be seen that the percentage of the BC symptoms group which reported no problems in four dimensions: self-care, usual activity, pain/discomfort and anxiety/depression was lower than that of the general population. In addition, no participants from the general population group reported the worst level of problems in any dimensions, while 1.5%, 3.8%, and 6.8% of the BC symptoms group indicated unable/severe problems in usual activity, pain/discomfort, and anxiety/depression, respectively.

**Table 3:** Comparison of proportions of the EQ-5D-5L self-reported health status of the Breast Cancer (BC) symptoms sample with the general population sample.

Level of problems	Mobility			Self-Care			Usual Activity			Pain/Discomfort			Anxiety/Depression		
	BC	GP	χ2 (df); P-value	BC	GP	χ2 (df); P-value	BC	GP	χ2 (df); P-value	BC	GP	χ2 (df); P-value	BC	GP	χ2 (df); P-value
No	90.9	89.0	11.95 (3); 0.008	92.4	98.0	13.20 (3); 0.004	78.0	86.7	20.88 (4); <0.001	27.3	57.3	62.95 (4); <0.001	18.2	63.3	147.19 (4); <0.001
Slight	3.8	9.2		3.8	1.5		13.6	11.5		51.5	38.5		47.7	29.8	
Moderate	3.0	1.5		3.0	0.2		5.3	1.7		13.6	3.1		15.9	6.5	
Severe	2.3	0.2		0.8	0.2		1.5	0.0		3.8	1.2		11.4	0.4	
Unable/ Extreme	0.0	0.0		0.0	0.0		1.5	0.0		3.8	0.0		6.8	0.0	

BC = breast cancer symptom; GP = general population

For the MANCOVA analysis, we included the educational level and monthly income as covariates because only these two characteristics differed significantly between the two groups. The results still showed significant overall differences in the QOL between women with BC symptoms and the general population (Wilks’ lambda of 0.85; p-value<0.001). A MANCOVA conducted on the health status yielded similar results (Wilks’ lambda of 0.82; p-value<0.001).

Further multiple linear regression analysis conducted only in the BC symptoms group showed that participants who lived in a rural area demonstrated higher social domain scores than they who did not live in a rural area. Concerning education, participants with the lowest educational levels (i.e. primary school and below) demonstrated lower scores (less favorable) on physical, social, and environmental QOL domains than participants who had college/university level of education, while only physical health scores were significantly different between the middle and the lowest levels of education. Participants in the lowest monthly income group demonstrated less favorable scores in physical, psychological, and environment QOL domains than participants in the highest monthly income group, while only the social domain score was significantly different between the middle and lowest-income groups. With respect to health status (EQ-5D-5L), participants who had the lowest educational level demonstrated significantly lower EQ-5D-5L index scores than participants who achieved higher educational level. We found that age had no significant association with QOL and health status of women with BC symptoms. Details can be seen in Table 4.

**Table 4:** Coefficients (B-values) from multiple linear regression analysis for quality of life and health status in women with Breast Cancer (BC) symptoms.

Predictors	Physical QOL			Psychological QOL			Social QOL			Environmental QOL			EQ-VAS			Index score		
	Coeff	SE	P-value	Coeff	SE	P-value	Coeff	SE	P-value	Coeff	SE	P-value	Coeff	SE	P-value	Coeff	SE	P-value
Group^a^																		
BC	-6.7	1.3	0.000	-4.8	1.4	0.001	-1.8	1.5	0.205	-2.0	1.3	0.141	-11.2	1.7	0.000	-0.2	0.0	0.000
Residence^b^																		
Urban	0.0	0.0	0.389	0.0	0.0	0.970	-0.1	0.0	0.139	0.0	0.0	0.695	-0.1	0.1	0.084	0.0	0.0	0.001
Age	-1.1	1.0	0.268	-2.2	1.1	0.041	-2.7	1.1	0.017	-1.3	1.0	0.198	-3.0	1.3	0.025	0.0	0.0	0.206
Education^c^																		
Middle	1.8	1.1	0.109	2.1	1.2	0.091	2.4	1.3	0.065	2.5	1.2	0.034	0.4	1.5	0.777	0.0	0.0	0.051
High	2.6	1.6	0.098	4.7	1.8	0.008	5.4	1.8	0.003	6.6	1.7	0.000	4.0	2.1	0.059	0.0	0.0	0.097
Income^d^																		
Middle	2.5	1.5	0.084	2.0	1.6	0.223	3.8	1.7	0.022	3.6	1.5	0.020	1.9	2.0	0.345	0.0	0.0	0.027
High	7.5	2.2	0.001	7.0	2.4	0.004	5.8	2.5	0.021	9.2	2.3	0.000	5.3	3.0	0.074	0.1	0.0	0.044
Constant	67.8	1.8	0.000	64.2	2.0	0.000	63.2	2.1	0.000	56.7	1.9	0.000	82.3	2.5	0.000	0.9	0.0	0.000

^a^General population is the reference

^b^Rural is the reference group

^c^Basic education level: primary school and below is the reference group

^d^Low monthly income is the reference group

## Discussion

To our knowledge, this is the first study that compared the QOL between Indonesian women with BC symptoms before the definitive diagnosis and Indonesian women in general. We found that the QOL of Indonesian women with BC symptoms was significantly lower than in the general Indonesian population, especially in the physical and psychological domain. They also reported more problems across all dimensions, namely mobility, self-care, usual activity, pain/discomfort and anxiety/depression. These findings were also maintained after correction for demographic differences. In addition, we found that education and monthly income were positively associated with the QOL and health status among the women with BC symptoms.

Previous studies among patients with BC reported that pain/discomfort and anxiety/depression are the most common symptoms reported. This might be associated with lower level of HRQOL among patients across different states of BC: after primary BC, during recurrence, and metastases [28–30]. Our study extended these previous results by adding that in the phase of pre-definite diagnosis, the similar results occurred: a higher percentage of reported problems in pain/discomfort and anxiety/depression. Note that because of the EQ-5D wording in the two aforementioned dimensions, we don’t know yet whether the women felt pain or discomfort and anxiety or depression. Further explorations might be needed to investigate whether participants have problems on only one or both conditions, e.g. pain or discomfort.

Concerning the group of women with BC symptoms, we found that higher levels of education and income were associated with more favorable physical, social, and environmental dimensions of QOL compared to those with lower levels of education. This finding is consistent with previous studies in other populations which demonstrated that both income [15, 31, 32] and education [33] level have a significant impact on QOL: the lower, the worse. It may be hypothesized that higher socio-economic and educational level of patients may lead to better access to information and health services; as a result, these individuals may have fewer problems and feel less uncertain.

Certain limitations of the current study should be considered. First, the sample of women with BC symptoms was obtained from only one area in Indonesia, West Java, that might not be a representative for the whole Indonesian archipelago. Second, the participants of the present study were women with BC symptoms who visited the hospital. It could be argued that these women were anxious enough about the symptoms they observed to enable them to visit the hospital, compared to the women who did not visit the hospital although they probably observed some BC symptoms. This might have biased the results. Third, the comparison group consisting of adult women from the general population, was not screened for the presence of any diseases. Therefore, it is possible that this group included a few participants with BC symptoms, a BC diagnosis, or BC survivors. This might have influenced the results, leading to an underestimation of the actual differences between the groups. Fourth, the choice of generic instruments: WHQOL-BREF and EQ-5D-5L, might be not sensitive enough to measure the QOL and health status of the patients compared to disease-specific instruments such as the European Organization for Research and Treatment of Cancer QOL Questionnaire-C30 (EORTC QLQ-C30) [34] and the Functional Assessment of Cancer Therapy-General (FACT-G) [35]. However, since the aim of the present study was to compare QOL of women with BC symptoms to women from the general population, which are less likely to have any BC symptoms, generic QOL instruments were considered as the best tool to serve this aim. Nevertheless, there are significant differences between the groups, which indicate worse QOL and health status in the group with BC symptoms. Fifth, although the EQ-5D-5L had been validated and used in breast cancer patients’ population in different countries across the world [25], this was not the case for Indonesia. So, it is not known for certain that the psychometric properties are supported accurately in the context of this study.

Future research might investigate other factors that may contribute to the QOL of women with BC symptoms, such as social support or physical activities, and find and evaluate effective ways to promote and improve their QOL. Studies might evaluate strategies carried out by healthcare providers and professionals, e.g., physicians, nurses, psychologists, or community health workers to increase their compliance and reduce physical and psychological problems, such as provision of individualized information, symptom management, counseling, or psychosocial interventions.

## Conclusions

Our study showed that Indonesian women with BC symptoms before the definitive diagnosis reported lower physical and psychological QOL and more pain/discomfort and anxiety/depression compared to the Indonesian women in general. Awareness and support for them from the medical field might improve these aspects of QOL.

## Abbreviations

BC: Breast cancer
EQ-5D-5L: European Quality of Life-5 Dimensions-5 Levels
HICs: High-income countries
LMICs: Low-middle income countries
QOL: Quality of life
WHOQOL-BREF: World health organization Quality of life BREF
